# The *MGMT* promoter single-nucleotide polymorphism rs1625649 had prognostic impact on patients with *MGMT* methylated glioblastoma

**DOI:** 10.1371/journal.pone.0186430

**Published:** 2017-10-16

**Authors:** Chih-Yi Hsu, Hsiang-Ling Ho, Shih-Chieh Lin, Tiffany Dai-Hwa Ho, Donald Ming-Tak Ho

**Affiliations:** 1 Department of Pathology and Laboratory Medicine, Taipei Veterans General Hospital, Taipei, Taiwan; 2 School of Medicine, National Yang-Ming University, Taipei, Taiwan; 3 Department of Computer Science and Department of Statistics, Duke University, Durham, United States of America; 4 Department of Pathology and Laboratory Medicine, Cheng Hsin General Hospital, Taipei, Taiwan; Beijing Cancer Hospital, CHINA

## Abstract

Promoter methylation is the most significant mechanism to regulate O^6^-methylguanine-DNA-methyltransferase (*MGMT*) expression. Single-nucleotide polymorphisms (SNPs) in the *MGMT* promoter region may also play a role. The aim of this study was to evaluate the clinical significance of SNPs in the *MGMT* promoter region of glioblastoma. Genomic DNAs from 118 glioblastomas were collected for polymerase chain reaction (PCR) amplification. Sanger sequencing was used to sequence the *MGMT* promoter region to detect SNPs. The results were correlated with *MGMT* status and patient survival. Rs1625649 was the only polymorphic SNP located at the *MGMT* promoter region in 37.5% of glioblastomas. Homozygous rs1625649 (AA genotype) was correlated with a higher *MGMT* methylation level and a lower protein expression, but the result was not statistically significant. In patients with *MGMT* methylated glioblastoma, cases with homozygous rs1625649 (AA genotype) were significantly associated with a lack of MGMT protein expression and a better progression-free survival (PFS) than the cases with wild type rs1625649 (CC genotype) or heterozygous rs1625649 (CA genotype). The survival impact was significant in multivariate analyses. In conclusion, the *MGMT* promoter homozygous rs1625649 (AA genotype) was found to correlate with a better PFS in patients with *MGMT* methylated glioblastoma.

## Introduction

The combined usage of radiotherapy and temozolomide (TMZ) to treat glioblastoma (GBM) patients has improved the 2-year survival rate from 10.9% (radiotherapy alone) to 27.2% [1]. Similar improvements have also been observed in our institute: the 2-year survival rates of patients in the pre-TMZ era (1995–1999) and the TMZ era (2007/10–2011/08) were 10.5% and 25.6%, respectively. Methylation of the O^6^-methylguanine-DNA-methyltransferase (*MGMT*) gene promoter is the major prognostic factor for longer survival; it is also predictive of the benefit from TMZ chemotherapy [2]. *MGMT* genetic changes [3, 4], histone modifications [5, 6], p53 derangement [7, 8] and post-transcriptional regulation [9, 10] could also lead to *MGMT* silencing.

In our previous studies, the *MGMT* status tested using immunohistochemistry (IHC), methylation-specific PCR (MSP), quantitative real-time MSP (qMSP), and/or pyrosequencing all showed significant correlation with TMZ treatment response and patient survival [11–13]. However, the methylation status did not match with protein expression in 16.3% (15/92) of the cases [11]. This discrepancy could be due to mechanisms other than promoter methylation that control MGMT expression, such as *MGMT* genetic polymorphism.

Polymorphisms in the *MGMT* gene may affect the primary structure, expression and DNA repair activity of MGMT [14]. A meta-analysis suggested that Leu84Phe and Ile143Val in the *MGMT* gene are risk factors for cancer [15]. Although their functional/biological significance are not clear, several single-nucleotide polymorphisms (SNPs) exist in the *MGMT* promoter region [16]. Some important observations were made: rs1625649 was associated with allelic expression imbalance [17] and rs16906252 *MGMT* promoter SNP was associated with *MGMT* promoter methylation [18–21] and longer survival in GBM patients [20, 21]. Contrary to these observations, a study of cell lines showed rs16906252 increased *MGMT* transcription [22].

In this study, we intended to evaluate the clinical significance of SNP in the *MGMT* promoter region. We sequenced the *MGMT* promoter region to detect SNPs in a group of GBMs. The results of SNPs were correlated with *MGMT* status, clinical characteristics and the survival of patients.

## Materials and methods

### Patients

The study protocol was approved by the Institutional Review Board of Taipei Veterans General Hospital, Taiwan, R.O.C. (#2015-01-003BC). One hundred and eighteen (118) primary GBM patients who had received TMZ chemotherapy with concomitant radiotherapy were selected from the surgical pathology file of the Department of Pathology and Laboratory Medicine, Taipei Veterans General Hospital, Taiwan, from October, 2007 to September, 2013. The general data of the patients, including age, gender, Karnofsky performance status (KPS), date of surgery, extent of resection, history of radiotherapy and medication were retrieved from their medical records. A 25% or more increase in size of enhancing tumor or any new tumor on magnetic resonance imaging (MRI) was considered as progression according to the Macdonald criteria [23]. The results of the IHC assessing the MGMT protein expression and the qMSP analyzing the methylation level of MGMT promoter were from a previous study of ours [24]. A positive MGMT staining (IHC+) was defined to be the staining intensity of the majority of tumor cells similar to that of the adjacent endothelial cells [11]. Because the 0.1 cutoff is the most distinguishing point correlated with survival, samples with *MGMT* qMSP methylation level >0.1 were classified as positive [24]. qMSP is a quantitative assay and its sensitivity (0.6%) is better than that of pyrosequencing (~5%). It evaluates more CpG sites (9) than pyrosequencing does (4). For each patient, the original histopathology slides were reviewed to confirm the diagnosis. Appropriate section and tumor area were selected for SNP analysis. The data and samples were analyzed anonymously after a coding procedure.

### Genotyping for the *MGMT* promoter region SNP

The *MGMT* promoter region is located at chromosome 10q26 (chromosome position 131264447–131265603). Genomic DNA was isolated from paraffin-embedded tissue using the PicoPure DNA extraction kit (Applied Biosystems, Foster City, CA, USA). The BIOMED-2 protocol was used to screen the quality and amplifiability of the isolated DNA [25]. DNA was PCR-amplified using the primers listed in S1 Table. Sanger sequencing was performed on the PCR product to detect SNPs.

### PCR amplification and direct sequencing of *IDH1* and *IDH2*

The data of the R132H-mutant IDH1 IHC were from a previous study of ours [24]. *IDH1* sequencing was performed on 114 GBMs, 8 of which were IHC-positive and 106 of which were IHC-negative. *IDH2* sequencing was done on all the 55 IDH1-wildtype GBM, of which 47 patients were <55 years of age and 8 patients were ≥55 years of age.

The genomic regions containing the catalytic domain of *IDH1*, including codon 132, and of *IDH2*, including codon 172, were amplified by PCR with the annealing temperature at 60°C. Primers used for PCR amplification were listed as follows: *IDH1* forward primer 5’-GTTGGCGTCAAATGTGCCAC-3’ and reverse primer 5’-GCCAACATGACTTACTTGATCC-3’; *IDH2* forward primer 5’-AGCCCATCATCTGCAAAAAC-3’ and reverse primer 5’-CTAGGCGAGGAGCTCCAGT-3’. The polymerase chain reaction (PCR) amplicons were subjected to Sanger sequencing using the Genetic Analyzer 310 (Applied Biosystems, Foster City, CA, USA), and subsequently analyzed by Mutation Surveyor software V3.00 (Soft-Genetics, State College, PA).

### Statistical analysis

The Fisher's exact test or chi square test was used to compare the distribution of categorical variables. Differences in continuous variables were compared by Mann-Whitney or Kruskal-Wallis test. Progression-free survival (PFS) was measured from the date of surgery to the date of progression. Overall survival (OS) was measured from the date of surgery to the date of death or last follow-up. PFS and OS curves were plotted using Kaplan-Meier method, and their differences were calculated with the log-rank test. Cox regression model was used to adjust the influence of age, gender, KPS, extent of resection, bevacizumab treatment, and IDH1 status. *P*-values were derived from 2-tailed tests and *P* < 0.05 was considered significant.

## Results

### SNP of *MGMT* promoter region

Rs1625649 was the only polymorphic SNP found in the *MGMT* promoter region, with the rest being homozygous for the common allele. It occurred in 44 (37.3%) cases, 27 (22.9%) were heterozygous genotype (CA) and 17 (14.4%) were homozygous genotype (AA). No other polymorphic SNP was found, including rs16906252 identified by previous studies [20, 21].

### Correlation with *MGMT* status and clinical characteristics

The *MGMT* status and patients’ clinical characteristics stratified by rs1625649 genotypes are listed in Table 1, with the details listed in S2 Table. Except for *IDH1* R132H mutation, there was no other *IDH1* mutation or *IDH2* mutation. Despite not reaching statistical significance, GBMs with homozygous rs1625649 (AA genotype) had a higher *MGMT* methylation level, lower frequency of MGMT protein expression, as well as better PFS and OS than those with heterozygous rs1625649 (CA genotype) and those with wild type rs1625649 (CC genotype). Of the *MGMT* methylated tumors, AA genotype had a higher mean methylation level (23.56) than CA (3.44) and CC (8.90) genotype (*P* = 0.074, Table 2). The MGMT protein expression in the methylated tumors (qMSP+) was significantly different among rs1625649 genotypes (*P* = 0.039, Table 2), and it was entirely absent in those with homozygous rs1625649 (AA genotype; 0%).

**Table 1:** Association between rs1625649 genotypes, *MGMT* status and clinical characteristics.

	Total	rs1625649 genotypes			*P*
		CC	CA	AA	
Number of patients	118	74	27	17	
*MGMT* qMSP value	4.7 (2.4, 6.9)	4.3 (1.5, 7.1)	1.5 (0.4, 2.7)	11.1 (0.7, 21.5)	0.816
*MGMT* qMSP+ (>0.1)	56 (47.5%)	36 (48.7%)	12 (44.4%)	8 (47.1%)	0.932
MGMT IHC+	55 (46.6%)	33 (44.6%)	17 (63.0%)	5 (29.4%)	0.080
Age ≥ 50 years	73 (61.9%)	49 (66.2%)	14 (51.9%)	10 (58.8%)	0.405
Male gender	71 (60.2%)	42 (56.8%)	18 (66.7%)	11 (64.7%)	0.612
KPS ≥ 80	65 (55.1%)	37 (50.0%)	18 (66.7%)	10 (58.8%)	0.311
Gross total resection	97 (82.2%)	59 (79.7%)	23 (85.2%)	15 (88.2%)	0.639
Bevacizumab treatment	26 (22.0%)	15 (20.3%)	8 (29.6%)	3 (17.7%)	0.540
IDH1 IHC+	10 (8.5%)	7 (9.5%)	1 (3.7%)	2 (11.8%)	0.571
*IDH1* R132H mutation	8/114 (7.0%)	6/71 (8.5%)	1/27 (3.7%)	1/16 (6.3%)	0.707
Median PFS (months)	7.5	7.4	7.0	13.5	0.098
Median OS (months)	18.0	17.2	19.4	24.6	0.238

Data presented as N (%) or mean (95% confidence interval).

qMSP, quantitative methylation specific PCR; KPS, Karnofsky performance status; IDH1, isocitrate dehydrogenase 1; IHC, immunohistochemistry; PFS, progression-free survival; OS, overall survival.

**Table 2:** Association between rs1625649 genotypes and MGMT status subgrouped by promoter methylation (qMSP) and protein expression (IHC).

MGMT status		Total	rs1625649 genotypes			*P*
qMSP	IHC		CC	CA	AA	
Positive (>0.1)		56 (100%)	36 (100%)	12 (100%)	8 (100%)	
Mean (95% CI)		9.82 (5.33, 14.32)	8.90 (3.43, 14.37)	3.44 (1.18, 5.7)	23.56 (3.16, 43.96)	0.074
	IHC+	7 (12.5%)	3 (8.3%)	4 (33.3%)	0 (0%)	0.039
	IHC–	49 (87.5%)	33 (91.7%)	8 (66.7%)	8 (100%)	
Negative (≤0.1)		62 (100%)	38 (100%)	15 (100%)	9 (100%)	
Mean (95% CI)		0.01 (0, 0.01)	0.01 (0, 0.01)	0.01 (0, 0.02)	0 (0, 0.01)	0.709
	IHC+	48 (77.4%)	30 (79.0%)	13 (86.7%)	5 (55.6%)	0.197
	IHC–	14 (22.6%)	8 (21.1%)	2 (13.3%)	4 (44.4%)	

Data presented as N (%) or mean (95% confidence interval).

qMSP, quantitative real-time methylation specific PCR; IHC, immunohistochemistry.

### Correlation with survivals

**Fig 1:**
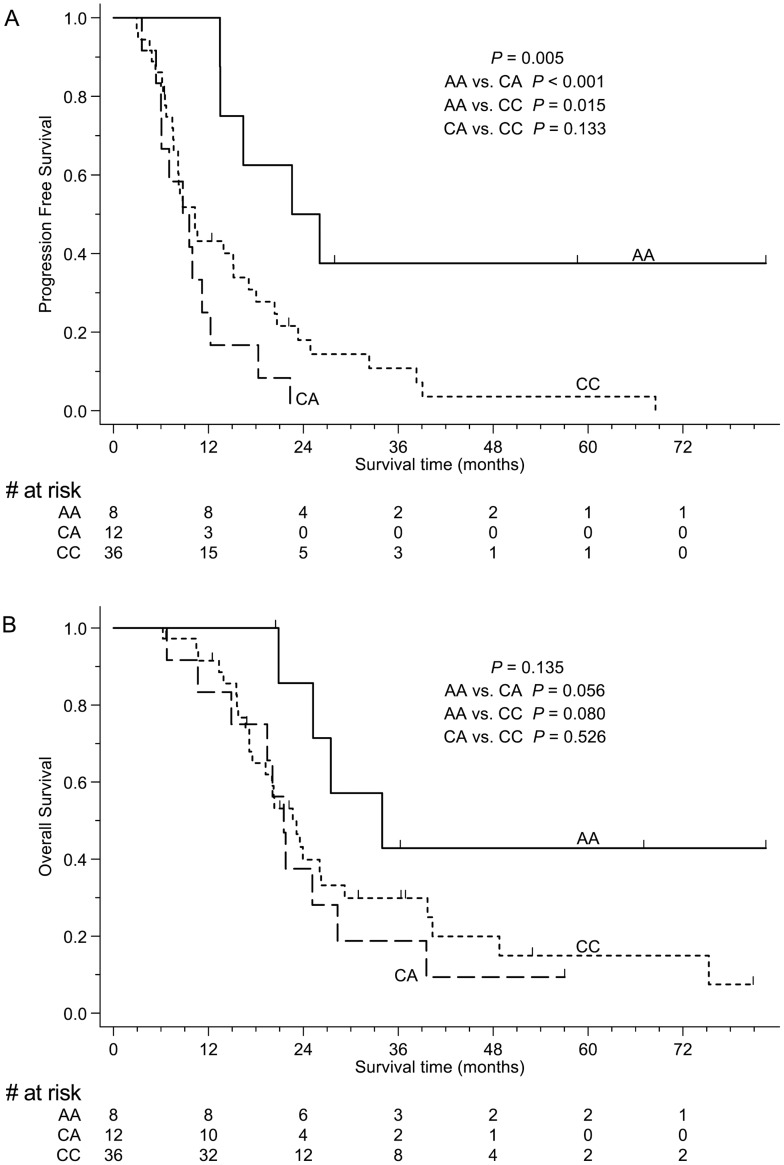
Survival curves stratified by rs1625649 genotypes in patients with *MGMT* methylated GBM. (A) Progression-free survival and (B) overall survival of the study cohort. Patients with homozygous rs1625649 (AA genotype) had longer progression-free survival than those with heterozygous (CA genotype) or wild type (CC genotype) rs1625649. However, the difference in overall survival did not reach statistical significance.

Among the patients with *MGMT* methylated GBM, those with homozygous rs1625649 (AA genotype) had longer PFS than those with heterozygous (CA genotype) or wild type (CC genotype) rs1625649 (Fig 1). The correlation remained statistically significant after the adjustment of age, gender, KPS, extent of resection, bevacizumab treatment and IDH1 status in multivariate analyses (Table 3). There was a similar trend in the OS, but their differences were not significant.

**Table 3:** Multivariate analyses of progression-free survival and overall survival stratified by rs1625649 genotypes in patients with *MGMT* methylated glioblastoma.

Genotype	N	Progression-free survival	Overall survival
CC	36	2.876 (0.050)	2.195 (0.189)
CA	12	5.835 (0.003)	3.328 (0.063)
AA	8	1 (reference)	1 (reference)

Data presented as Hazard Ratio (*P*). The influences of age, sex, Karnofsky performance status, extent of resection, bevacizumab treatment, and IDH1 status are adjusted.

## Discussion

This study showed that rs1625649 was the only polymorphic SNP in the promoter region of *MGMT* in Taiwanese GBM cases. The global minor allele frequency of rs1625649 in the dbSNP database is 0.403; its clinical significance is not available. In a study of lung cancer risk, rs1625649 appeared to have an additive interaction with smoking, and it was one of the four factors included in the best model of lung cancer risk [26]. A meta-analysis including 2504 cancer cases and 3494 controls revealed no significant association between rs1625649 and overall cancer risks [15].

SNPs in regulatory non-coding regions can influence DNA helical conformations, transcript splicing, protein binding, and the methylation status of CpG dinucleotides [27]. In a cell line study, rs1625649 alone could downregulate the *MGMT* promoter activity in the transfected glioblastoma cells [16]. In a study of 169 lung adenocarcinomas, rs1625649 was found to be modestly associated with *MGMT* methylation [28]. In our study of GBMs, despite not being statistically significant, there was an association between homozygous rs1625649 (AA genotype) and higher *MGMT* methylation level and lower MGMT protein expression (Table 1). Our observations are consistent with the results of previous studies [16, 28]. In the methylated cases, the MGMT protein expression had statistically significant correlations with the genotype, among which was an association between a complete absence of protein expression and the AA genotype (Table 2; *P* = 0.039). The correlation suggests that the homozygous (AA) genotype of rs1625649 had an enhancing effect on the lack of protein expression when methylation was present. On the other hand, the genotypes of the SNP did not have any significant effect on protein expression (*P* = 0.197) in the absence of methylation. The better PFS in cases with AA genotype could be interpreted as a consequence of the absence of MGMT protein expression, which was related to better response to TMZ treatment. The fact that better OS in cases with AA genotype did not reach statistical significance could be attributed to the small sample size. Given that *MGMT* promoter methylation is a clinically relevant predictor of TMZ chemotherapy and that the effect of rs1625649 on MGMT protein expression is related to the promoter methylation status, the assessment of SNP could be combined with the assessment of *MGMT* methylation for clinical use. Since MGMT protein expression was completely absent in the *MGMT* methylated cases with rs1625649 homozygous genotype (AA), a better TMZ chemotherapeutic response could be expected. As a result, TMZ monotherapy could be a treatment option for patients who have *MGMT* methylated tumor(s) with AA genotype and cannot receive radiotherapy.

Different from our study cohort, a study of 49 cases of grade III and IV gliomas did not show prognostic significance of rs1625649 [29]. The prognostic information of rs1625649 in *MGMT* methylated gliomas was, however, not provided [29]. In a study of metastatic colorectal cancer treated with oxaliplatin-based chemotherapy, homozygous rs1625649 (AA genotype) was associated with a worse PFS [30]. Further studies including more cases with similar tumor and treatment are needed to elucidate this issue.

Rs16906252 has been associated with *MGMT* promoter methylation [18–21]. The global minor allele frequency of rs16906252 is 0.025 in the dbSNP database. However, this SNP was mostly reported from the Western countries [18–21], and it has not been reported from the East Asian countries. It was also not found in our GBM cases. By comparing the protein expression level of the haplotypes with rs1625649 alone and that of the haplotypes with rs1625649 plus rs16906252, Xu et al. suggested that rs16906252 did not play a key role in the reduction of *MGMT* promoter activity [16].

In conclusion, rs1625649 was the only polymorphic SNP located in the *MGMT* promoter region in 37.5% of Taiwanese GBM cases. Among the patients with *MGMT* methylated GBM, those with homozygous rs1625649 (AA genotype) were associated with a lack of MGMT protein expression and a better PFS.

## Supporting information

S1 Table Primers for the single-nucleotide polymorphisms (SNPs) at MGMT promoter region. (PDF)

S2 Table Clinico-pathological features of the analyzed patients. (PDF)
