# Supplementary material for: The MGMT promoter single-nucleotide polymorphism rs1625649 had prognostic impact on patients with MGMT methylated glioblastoma
Source: PLoS One. 2017 Oct 16;12(10):e0186430. doi: 10.1371/journal.pone.0186430 (PMC5643071; doi:10.1371/journal.pone.0186430)
Supplement: S1 Table — (PDF) [file pone.0186430.s001.pdf]

**S1 Table. Primers for the single-nucleotide polymorphisms (SNPs) at MGMT promoter region.**

| Forward primer | Sequence | Reverse primer | Sequence | SNPs |
|---|---|---|---|---|
| *MGMT*-PE-F1 | AAG TGC CAC ACA CTG AGT GGC | *MGMT*-PE-R1 | GAG GAC ACT CTT CCC TGG AGC | rs180989103 |
| *MGMT*-PE-F2 | CTT AGG CTT CTG GTG GCT TGC | *MGMT*-PE-R2 | GTG TCC ACC AGA GAA GCT ATG | rs145103604, rs1711646, rs116191701 |
| *MGMT*-PE-F3 | CAT AGC TTC TCT GGT GGA CAC | *MGMT*-PE-R3 | GAC CAG CAG AGA CCC TGT CAG | rs61859810, rs186988330, rs117803818, rs74162154, rs115796770, rs138920082, rs60011905 |
| *MGMT*-PE-F4 | CTG ACA GGG TCT CTG CTG GTC | *MGMT*-PE-R4 | GTG CAA GCG ACC TGC CAC GTG | rs1625649, rs35322871, rs113813075 |
| *MGMT*-PE-F5 | CAC GTG GCA GGT CGC TTG CAC | *MGMT*-PE-R5 | CTT CGG CCG GTA CAA GCC GGG | rs79442343, rs34180180, rs189357135 |
| *MGMT*-PE-F6 | CCC GGC TTG TAC CGG CCG AAG | *MGMT*-PE-R62 | ACC GGG TCG GCG CAT GCC CTG T | rs112837630, rs34138162, rs1623007 |
| *MGMT*-PE-F7 | GCG CAG ACT GCC TCA GGC CC | *MGMT*-PE-R7 | GGG CAC GGG GAT GGC GGC GT | rs1623007, rs2782888, rs181536588 |
| *MGMT*-PE-F8 | ACG CCG CCA TCC CCG TGC CC | *MGMT*-PE-R8 | GAG GCT GCC ACC GTC CCG AG | rs16906252, rs113327489 |

SNP, single-nucleotide polymorphism.
