# Supplementary material for: The MGMT promoter single-nucleotide polymorphism rs1625649 had prognostic impact on patients with MGMT methylated glioblastoma
Source: PLoS One. 2017 Oct 16;12(10):e0186430. doi: 10.1371/journal.pone.0186430 (PMC5643071; doi:10.1371/journal.pone.0186430)
Supplement: S2 Table — (PDF) [file pone.0186430.s002.pdf]

S2 Table. Clinico-pathological features of the analyzed patients.

SNP 485	qMSP	MGMT IHC	AGE	Male	KPS 80	Total Resection	Avastin	IDH1 IHC	IDH1 seq	IDH2 seq	PFS	Prog	Survival time	Death
AA	1.523	0	69	0	1	1	0	0	0	0	13.47	1	20.87	1
CA	0.696	0	44	1	1	1	1	0	0	0	22.33	1	56.87	0
CC	52.302	0	63	0	1	1	0	0	0		20.67	1	26.07	1
CC	0	0	58	0	1	1	0	0	0		2.30	1	14.57	1
CA	0.673	0	53	1	1	1	0	0	0	0	18.30	1	28.33	1
CC	0	0	14	0	0	0	0	1	R132H		15.80	1	24.63	1
CC	0	1	65	1	0	1	0	0	0		1.47	1	6.50	1
AA	0	0	46	1	1	1	1	0	0	0	18.87	1	45.97	0
CC	0.51	0	67	1	1	0	0	0	0		10.30	1	16.70	1
CC	27.932	0	40	1	0	1	0	1	R132H		2.93	1	13.33	1
CC	1.328	0	58	1	1	0	0	0	0		4.57	1	23.13	1
CA	0	1	54	1	0	0	1	0	0	0	14.43	1	18.57	1
CC	18.903	0	37	0	1	0	0	0	0	0	7.60	1	23.93	1
CA	3.273	0	80	0	0	1	0	0	0		12.27	1	19.43	1
CA	0	1	9	0	1	1	1	0	0	0	5.60	1	14.60	1
CC	1.167	0	62	0	1	1	1	0	0		10.60	1	22.67	1
CA	0	1	48	1	0	1	0	0	0	0	1.10	1	8.70	1
CC	1.201	0	40	0	1	1	1	0	0	0	39.07	1	52.97	0
CC	0	1	41	1	0	1	0	0	0	0	1.83	1	3.57	1
CC	4.737	0	61	1	0	1	0	0	0	0	4.83	1	10.47	1
CC	0	1	54	0	0	1	0	0	0	0	4.67	1	10.73	1
CA	3.668	1	26	1	1	1	1	0	0	0	6.00	1	16.67	0
CC	0	1	15	0	0	1	1	0	0	0	2.50	1	4.87	1
CC	0	1	58	0	1	0	0	0	0		3.57	1	9.50	1
AA	0.592	0	63	1	1	1	1	0	0		22.57	1	25.23	1
CC	0	1	67	0	0	0	0	0	0		3.50	1	5.37	1
CC	0	1	25	1	0	1	1	0	0	0	3.10	1	7.23	1
CC	0	0	32	1	0	0	0	1	R132H		14.77	1	17.63	1
CC	29.253	0	56	0	1	0	0	0	0		15.17	1	20.23	1
CC	0	1	33	1	0	1	0	0	0	0	2.83	1	3.30	1
CC	5.136	0	79	0	0	1	0	0	0		18.03	1	19.23	1
CC	0	1	57	1	1	1	0	0	0		14.87	1	16.60	1
CC	40.332	0	60	0	1	1	0	0	0		20.37	1	29.23	1
CC	1.766	0	41	0	1	1	0	0	0	0	32.33	1	36.17	0

SNP 485	qMSP	MGMT IHC	AGE	Male	KPS 80	Total Resection	Avastin	IDH1 IHC	IDH1 seq	IDH2 seq	PFS	Prog	Survival time	Death
CC	1.111	0	83	1	0	1	0	0	0		13.90	1	13.90	1
CC	1.461	0	43	0	1	1	0	0	0	0	8.23	1	15.60	1
AA	0	1	71	0	0	1	1	0	0		5.00	1	12.00	1
CC	1.897	0	61	0	1	0	1	1	R132H		7.53	1	36.73	0
CA	0	1	72	1	1	1	0	0	0		29.27	1	30.63	0
CC	0	1	5	1	0	1	1	0	0	0	5.37	1	20.10	1
CA	0	1	70	0	1	1	0	0	0		14.83	1	22.43	1
CC	16.551	0	81	1	0	1	0	0			6.43	0	6.43	0
CC	0	1	60	1	0	1	0	0	0		22.63	1	34.30	1
AA	0	1	16	1	1	0	0	0	0	0	5.90	1	5.90	1
CC	0	1	65	1	1	1	0	0	0		2.23	1	4.10	1
CC	0	1	57	1	0	1	1	0	0		3.13	1	8.93	1
CC	0	1	7	0	0	1	1	0	0	0	2.97	1	13.30	1
CC	0	1	66	0	1	1	0	0	0		1.53	1	8.43	1
AA	11.516	0	35	0	1	1	0	0	0	0	27.77	0	36.07	0
CC	0	1	58	0	0	1	1	0	0		2.87	1	3.63	1
CC	0	1	55	0	0	1	0	0	0		19.30	1	22.00	0
CA	0.018	1	56	1	0	1	1	0	0		8.13	1	18.97	1
CC	1.012	1	73	1	0	1	0	0	0		21.97	0	22.00	0
CC	0	1	61	1	0	1	0	0	0		7.50	1	9.87	0
CC	0.54	0	40	1	0	0	1	0	0	0	10.30	1	20.83	0
CC	2.003	1	83	0	0	1	0	0	0		24.90	1	26.23	1
CA	0	0	36	1	1	1	0	1	R132H		24.67	0	33.20	0
CA	0	1	38	1	1	1	0	0	0	0	5.70	1	10.27	1
CC	0	1	55	0	0	0	1	0	0		19.60	0	19.60	0
CC	5.29	0	71	1	0	1	1	0	0		12.23	0	12.30	0
CC	70.385	0	66	0	1	1	0	0	0		15.13	1	30.77	0
CA	9.28	1	56	1	0	1	0	0	0		3.57	1	6.73	1
CC	0	1	54	1	1	1	0	0	0	0	5.07	1	10.60	1
CC	0.099	1	30	1	1	1	0	1	R132H		21.83	1	23.13	1
CC	0	1	62	1	1	1	0	0	0		3.17	1	14.80	1
CA	0	1	45	0	0	1	0	0	0	0	4.70	1	6.83	1
CA	6.47	1	30	1	0	1	1	0	0	0	8.77	1	10.67	1
AA	0	1	70	1	1	1	0	0	0		2.87	1	12.20	1
CC	0	0	47	1	1	1	1	0			10.43	1	18.27	0

SNP 485	qMSP	MGMT IHC	AGE	Male	KPS 80	Total Resection	Avastin	IDH1 IHC	IDH1 seq	IDH2 seq	PFS	Prog	Survival time	Death
CC	2.475	0	32	1	0	1	1	1			8.17	1	10.70	1
AA	38.868	0	49	0	1	1	0	1	R132H		82.30	0	82.30	0
CC	0.161	1	2	0	0	1	0	0	0	0	6.27	1	80.70	0
AA	71.038	0	13	1	0	1	0	0	0	0	13.50	1	27.47	1
CC	0	1	44	0	0	0	0	0	0	0	1.50	1	4.10	1
CA	3.674	0	61	1	1	1	0	0	0	0	5.37	1	20.10	1
AA	0	0	54	1	0	1	0	0	0	0	9.60	1	15.20	1
CC	0	0	76	1	1	1	0	0	0		5.40	1	6.33	1
CC	0	1	11	1	0	1	0	0	0	0	2.20	1	2.20	1
AA	8.859	0	61	1	0	1	0	0	0		26.07	1	33.97	1
CC	1.541	0	52	1	1	1	0	0	0	0	68.53	1	75.27	1
CA	0	1	38	1	1	1	0	0	0	0	2.83	1	22.77	1
CC	0.19	0	65	0	0	0	0	0	0		3.10	1	6.23	1
CC	0.666	0	56	0	1	1	0	0	0	0	8.40	1	15.53	1
CC	0	1	51	0	1	1	0	0	0	0	8.07	1	24.60	1
AA	0	0	53	1	0	1	0	0	0	0	12.83	1	15.33	1
CC	0.022	0	43	1	0	1	0	0	0	0	15.87	1	23.70	1
CA	0.666	0	33	1	1	1	0	0	0	0	11.20	1	21.53	1
AA	0	0	39	0	0	0	0	0	0	0	1.33	1	10.53	1
CC	0.596	0	56	1	0	1	0	0	0	0	5.30	1	17.57	1
AA	39.548	0	75	1	1	1	0	0	0		16.40	1	20.30	0
CC	2.333	0	70	0	1	1	1	0	0		38.30	1	48.80	1
CA	0.518	1	77	1	0	1	0	0	0		9.97	1	21.77	1
CA	0.962	0	46	0	1	1	0	0	0	0	9.57	1	25.13	1
CC	0	1	64	1	1	1	0	0	0	0	3.97	1	12.77	1
CC	0	0	12	1	0	0	0	0	0	0	7.17	1	14.03	1
CC	0	0	20	1	1	1	0	0	0	0	3.50	1	18.00	1
AA	0.02	1	78	1	0	1	0	0	0	0	4.03	1	24.63	1
CC	0	1	74	1	1	1	0	0	0		5.10	1	16.50	1
CC	1.106	0	61	1	1	1	0	0	0		23.33	1	40.33	1
CA	0.018	1	27	0	1	1	0	0	0	0	6.27	1	11.47	1
CC	1.449	0	68	1	1	1	0	0	0		6.67	1	39.70	1
CC	0.731	0	74	0	1	1	0	0	0		6.50	1	17.17	1
CA	0.097	0	55	1	1	1	1	0	0		1.20	1	12.07	1
CA	10.597	0	54	0	1	0	0	0	0	0	6.07	1	14.90	1

SNP 485	qMSP	MGMT IHC	AGE	Male	KPS 80	Total Resection	Avastin	IDH1 IHC	IDH1 seq	IDH2 seq	PFS	Prog	Survival time	Death
CC	0.038	1	85	1	0	1	0	0	0		2.30	1	2.97	1
CC	0	1	72	1	0	1	0	0	0		5.47	1	13.07	1
CA	0	1	60	1	1	1	0	0	0		4.07	1	15.87	1
CA	0	1	57	1	1	0	0	0	0		1.03	1	3.87	1
CC	0.247	0	84	0	1	1	0	0	0		6.13	1	20.03	1
CC	16.551	0	33	0	1	0	0	1	R132H		17.10	1	23.57	1
CC	1.199	0	53	1	1	1	0	0	0	0	8.73	1	17.17	1
CC	5.298	0	9	1	1	1	0	0	0	0	8.17	1	15.77	1
AA	0	1	53	1	1	1	0	0	0	0	3.60	1	11.83	1
AA	16.551	0	42	0	1	1	0	1			58.47	0	66.87	0
CC	1.03	0	60	1	1	1	0	0	0		7.43	1	20.33	1
CC	0.036	1	69	1	0	1	0	0	0	0	4.90	1	8.53	1
CA	0.002	1	7	0	0	0	1	0	0	0	7.00	1	21.53	1
CA	0.77	0	54	0	1	1	0	0	0	0	7.03	1	39.57	1

SNP, single-nucleotide polymorphism; qMSP, quantitative real-time methylation specific PCR; IHC, immunohistochemistry; KPS, Karnofsky performance status; Avastin, bevacizumab treatment; IDH1 seq, IDH1 sequencing; IDH2 seq, sequencing; PFS, progression-free survival; Prog, progress.
